# ZnSe Nanorods as Visible‐Light Absorbers for Photocatalytic and Photoelectrochemical H_2_ Evolution in Water

**DOI:** 10.1002/anie.201814265

**Published:** 2019-03-06

**Authors:** Moritz F. Kuehnel, Charles E. Creissen, Constantin D. Sahm, Dominik Wielend, Anja Schlosser, Katherine L. Orchard, Erwin Reisner

**Affiliations:** ^1^ Christian Doppler Laboratory for Sustainable Syngas Chemistry Department of Chemistry University of Cambridge Lensfield Road Cambridge CB2 1EW UK; ^2^ Department of Chemistry Swansea University Singleton Park Swansea SA2 8PP UK

**Keywords:** delafossite, hydrogen, photocatalysis, photocathode, zinc selenide

## Abstract

A precious‐metal‐ and Cd‐free photocatalyst system for efficient H_2_ evolution from aqueous protons with a performance comparable to Cd‐based quantum dots is presented. Rod‐shaped ZnSe nanocrystals (nanorods, NRs) with a Ni(BF_4_)_2_ co‐catalyst suspended in aqueous ascorbic acid evolve H_2_ with an activity up to 54±2 mmol${}_{H{}_{2}}$ g_ZnSe_^−1^ h^−1^ and a quantum yield of 50±4 % (*λ*=400 nm) under visible light illumination (AM 1.5G, 100 mW cm^−2^, *λ*>400 nm). Under simulated full‐spectrum solar irradiation (AM 1.5G, 100 mW cm^−2^), up to 149±22 mmol${}_{H{}_{2}}$ g_ZnSe_^−1^ h^−1^ is generated. Significant photocorrosion was not noticeable within 40 h and activity was even observed without an added co‐catalyst. The ZnSe NRs can also be used to construct an inexpensive delafossite CuCrO_2_ photocathode, which does not rely on a sacrificial electron donor. Immobilized ZnSe NRs on CuCrO_2_ generate photocurrents of around −10 μA cm^−2^ in an aqueous electrolyte solution (pH 5.5) with a photocurrent onset potential of approximately +0.75 V vs. RHE. This work establishes ZnSe as a state‐of‐the‐art light absorber for photocatalytic and photoelectrochemical H_2_ generation.

Artificial photosynthesis in which solar energy is stored in chemical fuels is a promising strategy for overcoming the temporal mismatch between renewable energy supply and demand.^1^ H_2_ is the most prominent example of a solar fuel as it can be generated by photoreduction of aqueous protons by a broad range of photocatalysts.^2^ Among the most active materials are chalcogenide nanocrystals based on CdS and CdSe.^3^ Despite the remarkable activities and stabilities shown by these materials,^4^ the toxicity and carcinogenic nature of cadmium represents a considerable obstacle for their wide‐spread application. Carbon‐based materials, such as carbon nitride,^5^ carbon dots,^6^ and conjugated organic polymers^7^ have recently been introduced as environmentally benign alternatives. While these materials are inexpensive and usually non‐toxic, their performances have yet to match those of Cd‐based photocatalysts to achieve high quantum yields for aqueous H_2_ production without precious and carcinogenic metals.

Here, we report ZnSe nanorods (NRs) as inexpensive Cd‐free light absorbers for efficient H_2_ evolution under visible‐light irradiation (Figure 1). The ZnSe NRs exhibit an activity approaching that of Cd‐based materials, even without an added co‐catalyst. Furthermore, we demonstrate that the high activity of the suspended ZnSe nanocrystals under sacrificial conditions can be translated to heterogeneous conditions by assembling a simple, precious‐metal‐free photoelectrode from ZnSe nanocrystals immobilized on *p*‐type delafossite CuCrO_2_.

**Figure 1:**
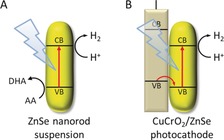
Schematic representation of the reported ZnSe nanorod photocatalyst system and its application for the construction of a noble‐metal‐free photocathode (CB: conduction band, VB: valence band, AA: ascorbic acid, DHA: dehydroascorbic acid).

ZnSe is a stable and inexpensive semiconductor with a direct bulk band gap of 2.7 eV,^8^ which enables absorption of near‐UV and some visible light. The conduction band (CB) is located at around −1.1 V vs. NHE (pH 0),^9^ providing ample driving force for the reduction of aqueous protons. Despite these favorable properties, ZnSe has received surprisingly little attention for solar fuel generation, unlike its cadmium analogues CdS and CdSe.^10^ Domen and co‐workers reported ZnSe/copper indium gallium selenide (CIGS) solid solution‐based photocathodes for H_2_ evolution^11^ with photocurrents up to 12 mA cm^−2^ at 0 V vs. the reversible hydrogen electrode (RHE) and onset potentials of +0.89 V vs. RHE.11b However, the complex photocathode assembly required a CdS charge extraction layer and a Pt proton‐reduction catalyst. While a number of reports have demonstrated the application of ZnSe‐based nanomaterials for photocatalytic dye degradation^12^ and water oxidation,^13^ only a few examples of Cd‐free ZnSe particles for photocatalytic H_2_ generation have been reported, all of which show low activity.^14^

We prepared ZnSe NRs by injecting trioctylphosphine/Se into an octadecane solution of zinc stearate at 300 °C, followed by a 25 min growth period.^15^ Surface modification of the as‐prepared stearate‐capped ZnSe NRs (ZnSe‐St) was achieved by ligand exchange with mercaptopropionic acid to give water‐soluble NRs (ZnSe‐MPA) and by reactive ligand removal with [Me_3_O][BF_4_] to give ligand‐free NRs (ZnSe‐BF_4_).^16^ Independent of the surface capping, the NRs are 5.2±0.6 nm in diameter and 30.0±4.8 nm long (aspect ratio 5.8±0.9), as determined from transmission electron microscopy (TEM, Figure S1 in the Supporting Information). Powder X‐ray diffraction (Figure S1 F) shows that the ZnSe NRs are obtained as a mixture of the zinc blende and wurtzite polymorphs, as previously observed with ZnSe nanorods synthesized by hot injection.^17^ The ZnSe NRs show UV‐visible light absorption up to about 440 nm (Figure S2 A) and two emission maxima separated by 0.097 eV in their photoluminescence (PL) spectra that can be attributed to differences in the band gaps of the two ZnSe polymorphs (Figure S2 B).^18^ Additional emissions at longer wavelengths likely result from trap states as previously observed with ZnSe nanocrystals.16a PL is reductively quenched by adding ascorbic acid (AA, Figure S2 C, D).

Figure 2 shows that ZnSe NRs are highly active photocatalysts for the reduction of aqueous protons to H_2_ under visible‐light irradiation (AM 1.5G, 100 mW cm^−2^, *λ*>400 nm) in the presence of AA. Under optimized conditions (pH 4.5, 0.4 m AA, 50 mg L^−1^ ZnSe, see Table S1 and Figure S3 in the Supporting Information for details on optimizing these parameters), ZnSe‐BF_4_ produced up to 33.6±2.0 mmol${}_{H{}_{2}}$ g_ZnSe_^−1^ h^−1^ (Figure 2 A). To further enhance the photocatalytic activity of the ZnSe NRs, Fe(BF_4_)_2_, Co(BF_4_)_2_, Ni(BF_4_)_2_, and K_2_PtCl_4_ were tested as co‐catalysts (Figure 2 A). Ni showed the highest performance increase to 54.3±1.9 mmol${}_{H{}_{2}}$ g_ZnSe_^−1^ h^−1^ at 20 μm, whereas K_2_PtCl_4_ quenched the photocatalytic activity almost completely. A low performance of Pt as a co‐catalyst has been previously observed with ligand‐free CdS.^19^ Pre‐formed Pt nanoparticles showed some activity, but still lower than without a co‐catalyst. Under the same conditions, ligand‐capped ZnSe‐MPA and ZnSe‐St NRs showed a lower H_2_ generation activity of 45.9±1.4 and 12.1±2.7 mmol${}_{H{}_{2}}$ g_ZnSe_^−1^ h^−1^, respectively (Figure 2 B). This observation agrees with our previous studies, which demonstrated an enhanced H_2_ evolution activity of CdS nanocrystals upon ligand removal.^20^ Under simulated full‐spectrum solar irradiation (AM 1.5G, 100 mW cm^−2^), ZnSe‐BF_4_ generates up to 149±22 mmol${}_{H{}_{2}}$ g_ZnSe_^−1^ h^−1^ and 95±27 mmol${}_{H{}_{2}}$ g_ZnSe_^−1^ h^−1^ in the presence and absence of Ni(BF_4_)_2_, respectively (Figure 2 C). The internal quantum yield (IQE) under monochromatic light (*λ*=400 nm) was 50.2±3.6 % (35.9±2.6 % external quantum yield, EQE; Supporting Information, Table S2).

**Figure 2:**
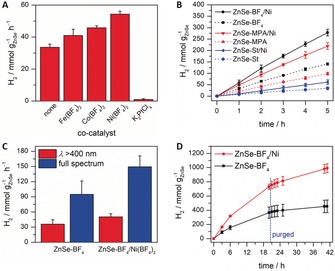
Photocatalytic H_2_ generation using aqueous ZnSe NRs. A) Ligand‐free ZnSe NRs in the presence of different co‐catalysts (3 h irradiation). B) Effect of the NR capping ligand. C) ZnSe‐BF_4_ under different irradiation spectra (1 h irradiation). D) Long‐term activity of ZnSe‐BF_4_ with the photoreactor being purged with N_2_ after 20 h. The cumulative amount of H_2_ is shown. Conditions unless stated otherwise: 50 mg L^−1^ ZnSe NRs, 0.4 m AA, pH 4.5, 20 μm Ni(BF_4_)_2_, 25 °C, 100 mW cm^−2^, AM 1.5G, *λ*>400 nm.

Long‐term experiments using ZnSe‐BF_4_ showed that H_2_ production is sustained over more than 40 h with a gradual decrease in rate (Figure 2 D). This decreasing activity is likely due to accumulation of dehydroascorbic acid (DHA) in solution. Photodegradation of ZnSe is only marginal, since separating ZnSe‐BF_4_ NRs after 20 h and re‐dispersing them in a fresh AA solution largely restored the activity (some material is lost during separation). In contrast, adding fresh ZnSe NRs had no effect on the activity (Supporting Information, Figure S4). Previous work has shown that the AA oxidation product DHA can inhibit the photocatalytic H_2_ production.^21^ UV/Vis spectra before and after prolonged irradiation show no degradation apart from an increase in scattering resulting from particle aggregation (Figure S5). Post‐catalysis TEM confirms the formation of aggregates with aspherical nanocrystalline features (Figure S6). Inductively‐coupled plasma optical emission spectroscopy (ICP‐OES) of ZnSe‐BF_4_/Ni isolated after 3 h irradiation showed an incorporation of 8.5±2.3 Ni atoms per ZnSe NR (<1 % of total added Ni), suggesting in situ formation of a heterogeneous Ni‐based catalyst on the NR surface.^22^ No H_2_ was generated without ZnSe, in the dark, or without an electron donor (Supporting Information, Table S3).

These data demonstrate that ZnSe‐BF_4_ NRs are efficient and stable light absorbers for aqueous H_2_ production, considerably outperforming previous Cd‐free ZnSe photocatalysts despite their blue‐shifted absorption spectrum compared to CdS. Previous reports have shown that a Pt/ZnO‐ZnSe nanocomposite generated 3 mmol${}_{H{}_{2}}$ g^−1^ h^−1^ under UV irradiation with 2.54 % EQE,14a and CoP‐decorated ZnSe nanobelts produced <1 mmol${}_{H{}_{2}}$ g^−1^ h^−1^ under visible‐light irradiation.14b However, the photocatalytic activities of ZnSe‐BF_4_ (33.6±2.0 mmol${}_{H{}_{2}}$ g_ZnSe_^−1^ h^−1^, 25.9±1.2 % EQE) and ZnSe‐BF_4_/Ni (54.3±1.9 mmol${}_{H{}_{2}}$ g_ZnSe_^−1^ h^−1^, 35.9±2.6 % EQE) approach those of Cd‐based photocatalysts^23^ such as Cd_0.25_Zn_0.75_Se/CoP (45.1 mmol${}_{H{}_{2}}$ g^−1^ h^−1^).14b CdSe quantum dots (QDs) combined with a Ni catalyst were shown to produce H_2_ with an IQE of 36±10 %.^24^ Higher performances were reported for CdS with different co‐catalysts^25^ such as MoS_2_ (96.7 mmol${}_{H{}_{2}}$ g^−1^ h^−1^, 46.9 % EQE),25a Ni_2_P (1200 mmol${}_{H{}_{2}}$ g^−1^ h^−1^, 41 % EQE),25b and Pt/PdS (29.2 mmol${}_{H{}_{2}}$ g^−1^ h^−1^, 93 % EQE).25c Without a co‐catalyst, up to 41 mmol${}_{H{}_{2}}$ g^−1^ h^−1^ 26 and 2.8 % EQE^27^ were reported for CdS, and 239 mmol${}_{H{}_{2}}$ g^−1^ h^−1^ 28 and 65.7 % EQE^29^ for Cd_*x*_Zn_1−*x*_S. Cd‐free alternatives such as CuInS_2_‐ZnS,^30^ carbon nitride,^31^ conjugated polymers,^32^ triazine frameworks,^33^ and polymer dots^34^ generally show much lower activities, although a recently reported NaCl/KCl‐treated carbon nitride/Pt material achieved up to 60 % EQE.^35^

Having established a good performance and stability of ZnSe nanorods for photocatalytic H_2_ production, even in the absence of an added co‐catalyst, we aimed to eliminate the sacrificial electron donor AA. The production of low‐value H_2_ gas at the expense of a sacrificial electron donor is not sustainable unless the electron donor is freely available, for example by photoreforming waste.^19^, ^36^ Instead, a nanocrystal‐sensitized photocathode can be assembled, where the nanocrystal provides electrons for the photocatalysis and a *p*‐type semiconductor accepts the photogenerated holes, replacing the chemical electron donor. Such systems enable overall water splitting through coupling with a photoanode for water oxidation.^37^

To this end, we immobilized ZnSe‐BF_4_ NRs on a CuCrO_2_ electrode. CuCrO_2_ is a wide‐band‐gap semiconductor (*E*_g_≈3.1 eV), which crystallizes in a delafossite‐type structure. Previous work has shown that modification of CuCrO_2_ with an organic dye and a nickel bis(diphosphine) catalyst enabled visible‐light‐driven proton reduction in aqueous solution.^38^ The characteristic high hole mobility, *p*‐type conductivity, and straightforward synthesis from abundant materials using solution processing techniques make CuCrO_2_ a suitable candidate for the coupling with ZnSe in a hydrogen‐generating photocathode.

The ZnSe nanorods were immobilized by drop‐casting (8 μL cm^−2^, 1.66 mg mL^−1^, acetonitrile) directly onto CuCrO_2_ electrodes (thickness approx. 300 nm, see Figure S7 in the Supporting Information; 13.4 μg ZnSe cm^−2^). EDX spectra confirmed an even distribution over the electrode surface (Figure S8). UV/Vis spectra of ZnSe‐modified CuCrO_2_ feature the characteristic absorptions of both CuCrO_2_ and ZnSe (Figure S9). Linear‐sweep voltammograms and chronoamperograms of ZnSe‐modified electrodes show enhanced photocurrents compared to the bare CuCrO_2_ electrode, with an onset potential of approximately +0.75 V vs. RHE (Figure 3), indicating the ability of photoexcited ZnSe nanorods to inject holes (*E*_VB,ZnSe_=1.6 V vs. RHE) into the valence band of CuCrO_2_ (*E* ${}_{\mathrm{VB},\mathrm{CuCrO}{}_{2}}$ =1.0 V vs. RHE).^38^ Controlled potential photoelectrolysis (CPPE; Supporting Information, Figure S10) confirmed that the highly reducing CB_ZnSe_ electrons are used to reduce aqueous protons to H_2_. CPPE with a CuCrO_2_|ZnSe electrode maintained at *E*_app_=0 V vs. RHE and illuminated from the front side (100 mW cm^−2^, AM 1.5G, *λ*>400 nm) produced 35±7 nmol H_2_ over the course of 4 h with a Faradaic efficiency (FE) of 7±2 % (Table S5). Bare CuCrO_2_ produced no detectable H_2_, confirming the essential role of ZnSe in this system. The high dark current, as previously reported for CuCrO_2_,^38^ and dissolved H_2_ which is not sufficiently accounted for in low current‐generating systems^39^ both contribute to the modest FE. Adding Ni^2+^ as a co‐catalyst increases the overall H_2_ production yield, corresponding well to photocatalysis results (Supporting Information, Figure S11 and Table S5). Incident photon‐to‐current efficiency measurements showed an increased current in the 400–440 nm region for CuCrO_2_|ZnSe electrodes compared to bare CuCrO_2_, confirming the role of ZnSe NRs in this photocathode (Figure S12).

**Figure 3:**
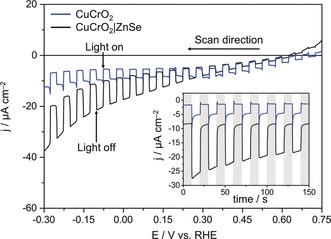
Linear‐sweep voltammograms under chopped light illumination for CuCrO_2_ (blue) and CuCrO_2_|ZnSe (black) electrodes, and chronoamperograms (inset) of the same electrodes at *E*_app_=0 V vs. RHE. Shading indicates dark chops. Conditions: Aq. Na_2_SO_4_ (0.1 m, pH 5.5), room temperature, 100 mW cm^−2^, AM 1.5G, *λ*>400 nm, scan rate 5 mV s^−1^. The photocurrent density was adjusted for an electrode area of 0.25 cm^2^.

H_2_‐generating QD‐sensitized photocathodes in the absence of a co‐catalyst have shown photocurrents of −60 μA cm^−2^ at 0.3 V vs. RHE with mercaptoacetic‐acid‐modified CdSe on NiO^40^ and −180 μA cm^−2^ at 0.5 V vs. RHE using a phenothiazine hole‐accepting ligand with CdSe on NiO.^41^ CuCrO_2_|ZnSe photoelectrodes generated −10 μA cm^−2^ at 0 V vs. RHE, comparable to the photocurrents observed with a molecular dye/catalyst assembly.^38^ The low photocurrent can be partly attributed to low light absorption, but the dominant limiting factor is likely a non‐ideal interface between CuCrO_2_ and the ZnSe NRs. This results in high charge recombination, limiting the number of electrons available for catalysis. Adding a H_2_ evolution co‐catalyst therefore only results in a small activity enhancement. Although this performance does not match that of the corresponding Cd‐based systems yet, it does demonstrate that the ZnSe NR photocatalyst can operate in the absence of a sacrificial reagent and in a photoelectrochemical cell. We expect future improvements for the integration of ZnSe into electrodes through CuCrO_2_ nanostructuring and ligand engineering to improve the CuCrO_2_/ZnSe interface,^40^, ^41^, ^42^ alternative assembly methods,^43^ and the integration of molecular catalysts,^44^ especially for CO_2_ reduction,16a making use of the highly reducing CB of ZnSe.

In summary, we have demonstrated that ZnSe nanorods are efficient light‐absorbers for solar‐driven H_2_ production, even without an added hydrogen‐evolution co‐catalyst. Their performance already approaches that of Cd‐containing quantum dots without exhibiting their carcinogenicity, highlighting the potential of designing novel inorganic materials for efficient photocatalysis. We showed that the ZnSe nanorods can also be integrated into photoelectrochemical cells, which paves the way to closed‐cycle solar fuel synthesis and we also envision its use in organic photoredox catalysis and photoreforming of waste and pollutants in future development.

## Conflict of interest

The authors declare no conflict of interest.

## Supporting information

As a service to our authors and readers, this journal provides supporting information supplied by the authors. Such materials are peer reviewed and may be re‐organized for online delivery, but are not copy‐edited or typeset. Technical support issues arising from supporting information (other than missing files) should be addressed to the authors.

Supplementary
