# Supplementary material for: ZnSe Nanorods as Visible‐Light Absorbers for Photocatalytic and Photoelectrochemical H2 Evolution in Water
Source: Angew Chem Int Ed Engl. 2019 Mar 6;58(15):5059–63. doi: 10.1002/anie.201814265 (PMC6492148; doi:10.1002/anie.201814265)
Supplement: Supplementary file 1 — Supplementary [file ANIE-58-5059-s001.pdf]

Angewandte Chemie
Eine Zeitschrift der Gesellschaft Deutscher Chemiker

Supporting Information

ZnSe Nanorods as Visible-Light Absorbers for Photocatalytic and Photoelectrochemical H_2 Evolution in Water

*Moritz F. Kuehnel[+], Charles E. Creissen[+], Constantin D. Sahm[+], Dominik Wielend, Anja Schlosser, Katherine L. Orchard, and Erwin Reisner**

anie_201814265_sm_miscellaneous_information.pdf

Experimental Section

Materials. Zinc stearate (purum, Sigma-Aldrich), octadecane (99 %, Sigma-Aldrich), trioctylphosphine (90 %, Sigma-Aldrich), selenium powder (99 %, Sigma-Aldrich), *n*-heptane (99 %, Aldrich), methanol (99.8 %, Fisher-scientific), acetone (laboratory reagent grade, Fisher-scientific), 1-butanol (99 %, Alfa Aesar), trimethyloxonium tetrafluoroborate (96 %, Sigma-Aldrich), tetramethyl ammonium hydroxide pentahydrate (99 %, Sigma-Aldrich), 3-mercaptopropionic acid (MPA, ≥99 %, Sigma-Aldrich), L-ascorbic acid (99 %, Sigma-Aldrich), $Fe(BF_4)_2 \cdot 6\,H_2O$ (97 %, Sigma-Aldrich), $Co(BF_4)_2 \cdot 6\,H_2O$ (99 %, Sigma-Aldrich), $Ni(BF_4)_2 \cdot 6\,H_2O$ (99 %, Acros) and K_2PtCl_4 (99.9 %, Sigma-Aldrich), Pt colloids (3 nm, 1000 ppm in water, Sigma-Aldrich) were used as received. Anhydrous solvents were purchased from Acros Organics with the following purities: $CHCl_3$ (99.9 %), *N*,*N*-dimethylformamide (DMF, 99.8 %), acetonitrile (ACN, 99.9 %). $Cu(acetate)_2 \cdot H_2O$ (ACROS Organics, ACS reagent), $Cr(NO_3)_3 \cdot 9H_2O$ (Sigma-Aldrich, ≥99 %), and triethanolamine (Sigma-Aldrich, ≥99.5 %) were used for $CuCrO_2$ film preparation. ITO-coated glass sheets (Vision Tek Systems Ltd., R = 12 Ω cm^{-2}, thickness of 1.1 mm) were cut into 3 × 3 cm^2 slides then scored into 1 × 1.5 cm^2 before use. All aqueous experimental solutions were prepared with distilled water and all aqueous analytical samples were prepared with ultrapure water (DI water; Milli-Q®, 18.2 MΩ cm).

ZnSe-St.[1] A zinc precursor solution was prepared by mixing zinc stearate (632 mg, 1.00 mmol) and octadecane (8 g) in a 50 mL three-necked flask and degassed at 50 °C for 2 h before raising the temperature to 300 °C under N_2. A selenium precursor solution was prepared by dissolving Se powder (79.0 mg, 1.00 mmol) in trioctylphosphine (2.5 mL) under N_2 at 50 °C for 50 min. The Se solution was then heated to 100 °C and quickly injected into the stirred Zn solution (500 rpm) with a glass syringe. After stirring the reaction mixture for 25 min at 300 °C, it was quenched by placing the flask into a cold oil bath. When a temperature of 55 °C was reached, *n*-heptane (40 mL) was quickly added to prevent octadecane solidification. The mixture was divided into 4 equal parts, and to each part methanol (10 mL) was added to precipitate ZnSe-St followed by adding acetone (2x10 mL) to form a single phase. Particles were separated by centrifugation (6,500 rpm, 8 min) and washed by repeated dissolution in $CHCl_3$ (5 mL), precipitation with methanol (15 mL), and centrifugation (6,500 rpm, 8 min). The precipitate was mechanically separated from the tube walls, dispersed in 1-butanol (10 mL), sonicated for 10 min and separated by centrifugation (6,500 rpm, 8 min). Residual butanol was removed by repeatedly adding methanol (10 mL), sonicating for 10 min and centrifugation (6,500 rpm, 8 min). Finally, the washed ZnSe-St was dissolved in $CHCl_3$ (3-4 mL) and centrifuged (6,500 rpm, 8 min), resulting in a cloudy upper layer, a clear middle layer and some precipitate on the bottom. The clear middle solution was collected and stored as stock solution at 4 °C.

ZnSe-BF₄.[2] ZnSe-St (6-10 mL stock solution) were placed in a Schlenk flask and the solvent was removed *in vacuo*. The residue was dissolved in anhydrous $CHCl_3$ (3.0 mL) and anhydrous DMF (0.20 mL) under N_2. $[Me_3O][BF_4]$ (1.0 M in anhydrous MeCN, 3-5 mL) was added dropwise, causing $ZnSe\text{-}BF_4$ to precipitate. The mixture was centrifuged (10,000 rpm, 10 min), the supernatant discarded, and precipitate re-dispersed in anhydrous DMF (3-4 mL), followed by sonication (20 min) and centrifugation (10,000 rpm, 10 min). The clear supernatant was degassed by two freeze-pump-thaw cycles and stored under N_2 at 4 °C.

ZnSe-MPA.[3] Tetramethyl ammonium hydroxide (TMAOH, 25 % in water, 1 mL) was added to a solution of mercaptopropionic acid (0.75 mL) in 1:1 $CHCl_3$/MeOH (15 mL) and the pH was adjusted to 11 by adding solid TMAOH (approx. 16 scoops). Under N_2, this solution was added to ZnSe-St (3 mL stock solution) and stirred at 300 rpm for 70 h in the dark. Subsequently, acetone (50 mL) was added and ZnSe-MPA was separated by centrifugation (6,500 rpm, 8 min). The precipitate was repeatedly suspended in acetone (5-10 mL), sonicated (5 min), and separated by centrifugation (6,500 rpm, 8 min). Finally, solid ZnSe-MPA was left to dry in air for 1 min and dissolved water (1.0 mL). The resulting clear solution was degassed by two freeze-pump-thaw cycles and stored under N_2 at 4 °C.

Powder X-ray diffraction (XRD). XRD was conducted using an PANalytical Empyrean Series 2 instrument using CuK_α irradiation. 100 µL ZnSe nanorod stock solution were drop-casted onto a Si wafer and dried under vacuum.

Inductively-coupled plasma-optical emission spectroscopy (ICP-OES). The concentration of all ZnSe nanorod stock solutions was determined by ICP-OES. Measurements were carried-out by the Microanalysis Services, Department of Chemistry, University of Cambridge using a Thermo Scientific iCAP 7400 spectrometer. Samples were digested in HNO_3 and diluted with ultrapure water to 1-10 ppm analyte concentration, and the Zn^{2+} concentration was determined. Blank samples of diluted HNO_3 were recorded as background. The ZnSe concentration was calculated from the determined Zn^{2+} concentration assuming an ideal 1:1 Zn:Se stoichiometry. $CuCrO_2$|ZnSe loading values were determined in triplicate, along with diluted HNO_3 blanks and bare $CuCrO_2$ electrodes, by digestion of a 1 cm^2 electrode in HNO_3 (1 mL) overnight and dilution to 10% v/v in Milli-Q® H_2O. Both Se and Zn content were determined for these samples.

Transmission electron microscopy (TEM). TEM images were collected using a FEI Philips Tecnai F20-G2 TEM, operating at an accelerating voltage of 200 kV located at the Electron Microscopy Suite of the Cavendish Laboratory, University of Cambridge. ZnSe-St stock solutions were diluted 1:1,000 in $CHCl_3$, drop-casted onto Cu grids and dried in air. ZnSe-BF_4 stock solutions were diluted 1:5,000 in DMF, drop-casted onto Cu grids and dried under vacuum. The grid was subsequently washed 10 times with $CHCl_3$ and dried in air. Post-catalysis TEM images were collected using a Thermo Scientific (FEI) Talos F200X G2 TEM, operating at an accelerating voltage of 200 kV located at the Department of Chemistry, University of Cambridge. ZnSe-NRs were precipitated after photocatalysis (3 h) via centrifugation, re-dispersed in DMF (2 mL), diluted (1:25) in H_2O and drop-casted onto holey-carbon coated Cu grids.

Scanning electron microscopy (SEM). SEM images were recorded using a Tescan MIRA3 FEG-SEM. Energy-dispersive X-ray (EDX) spectra were recorded with an Oxford Instruments Aztec Energy X-maxN 80 EDX system (20 kV, 15 mm working distance).

Photocatalytic H_2 generation. A ZnSe-NR stock solution (ZnSe-BF_4, ZnSe-MPA or ZnSe-St, 6.39 mg ZnSe mL^{-1}, 15.6 µL) and co-catalyst stock solution (2 mM aq. $Ni(BF_4)_2 \cdot 6H_2O$, 20 µL) were diluted in aq. ascorbic acid (0.4 M, pH adjusted to 4.5 with NaOH) to a total volume of 2.00 mL, to give a ZnSe concentration of 50 mg L^{-1}. The mixture was then added to a Pyrex photoreactor containing a stirrer bar (7.91 mL internal volume), sealed with a rubber septum and purged with N_2 (2 % CH_4) for 10 min in the dark. The photoreactor was then thermostated with a water circulator at 25 °C, stirred at 600 rpm and irradiated by a solar light simulator (Newport Oriel, 100 mW cm^{-2}) equipped with an air mass 1.5 global filter (AM 1.5G). IR irradiation was filtered with a water filter

(10 cm path length). Where indicated, UV irradiation was filtered with a 400 nm long pass filter (UQG Optics). Generated H_2 was quantified through periodic headspace gas analysis (50 µL) by gas chromatography.

For long-term stability tests, photocatalysis experiments were performed for 21 h, followed by either purging with N_2 (2 % CH_4) or centrifugation (10 min, 10,000 rpm). The separated precipitate was re-suspended in its original supernatant or in fresh aq. AA (0.4 M, pH 4.5). Alternatively, fresh ZnSe-BF_4 stock solution (6.39 mg ZnSe mL^{-1}, 15.6 µL) was added to used supernatant. All samples were sealed, purged with N_2 (2 % CH_4) for 10 min in the dark and irradiation was continued as stated above.

Gas chromatography analysis. Gas chromatography was carried out on either an Agilent 7890A gas chromatograph using a thermal conductivity detector and a HP-5 column (kept at 45 °C) and N_2 as the carrier gas or a Shimadzu Tracera GC2010 Plus gas chromatograph using a barrier ionization discharge (BID) detector and a molsieve column (kept at 130°C) with He as the carrier gas. Methane (2 % CH_4 in N_2, BOC) was used as internal standard after calibration with different mixtures of known CH_4/H_2/N_2 compositions.

External quantum efficiency (EQE). Photocatalysis samples were prepared as stated above, but using an airtight, flat-sided quartz cuvette (1 cm path length) as the photoreactor. The cuvette was purged with N_2/CH_4 (2 %) and irradiated with monochromatic light (λ = 400±5 nm, I = 1.0 mW cm^{-2}, A = 0.20 cm^2) using an LOT Quantum Design MSH-300 monochromator. Aliquots of headspace gas were taken periodically and analyzed by gas chromatography. The EQE was calculated according to equation (1).

$$\text{EQE (\%)} = \frac{2n \times N_A \times h \times c}{t_{\text{irr}} \times \lambda \times I \times A} \times 100 \qquad (1)$$

Where n is the amount of H_2 produced per time, N_A is Avogadro's constant, h is the Planck constant, c is the speed of light, t_{irr} is the irradiation time, λ is the irradiation wavelength, I is the irradiation intensity and A is the irradiated area. The internal quantum yield (IQE) was calculated by dividing the EQE by the extinction at 400 nm.

Determination of co-catalyst attachment. After 3 h photocatalytic H_2 production at standard conditions (*vide supra*), the reaction mixtures of 3 experiments were combined, transferred to a centrifuge tube and centrifuged for 10 min at 10,000 rpm. The precipitate was dissolved in conc. HNO_3 (2 mL) and diluted to 10 mL with ultrapure water. By ICP-OES, the Ni:Se ratio was found to be 0.450 ng Ni per µg Se. Using the ZnSe bulk density of 5.27×10^6 g m^{-3}, the weight of an individual ZnSe-NR (5.2±0.6 nm diameter, 30.0±4.8 nm long) was estimated at $3.36\pm0.92\times10^{-18}$ g, corresponding to a Ni content of $8.26\pm2.27\times10^{-22}$ g or 8.5±2.3 Ni atoms per NR.

UV−Vis spectroscopy. UV−Vis spectra were recorded on a Varian Cary 50 UV−Vis spectrophotometer using quartz glass cuvettes (1 cm path length). Spectra of electrodes were recorded in transmission mode with a blank ITO-coated glass background.

Preparation of $CuCrO_2$|ZnSe electrodes. $CuCrO_2$ electrodes were prepared directly on ITO-coated glass slides, which were previously cleaned (sonication in isopropanol, ethanol, and acetone, 15 min) and dried at 100 °C. A solution of Cu(acetate)$_2\cdot H_2O$ (0.2 M), $Cr(NO_3)_3\cdot 9H_2O$ (0.2 M), and triethanolamine (0.2 M), was prepared in absolute ethanol and stirred for 15 h before spin coating

on ITO slides (Laurell WS-650MZ spin coater, 1500 rpm, 15 s, 3000 rpm s^{-1} acceleration, 0.4 mL volume). Samples were annealed in a chamber furnace (Carbolite Gero) to 400 °C with a ramp rate of 10 °C min^{-1} for 45 min. Spin-coating and annealing steps repeated for a total of 3 layers. A tube furnace fitted with a quartz tube, end seals, and insulation plugs (Carbolite Gero) was used to anneal samples to 600 °C with a 5 °C min^{-1} ramp rate for 45 min under Ar flow (150 SCCM). ZnSe was immobilized through dropcasting 8 µL cm^{-2} of a stock solution (1.66 mg/mL, acetonitrile) and drying in air. $CuCrO_2$|ZnSe photoelectrodes were used directly after preparation.

Photoelectrochemical measurements. Photoelectrochemical measurements were conducted in a one-compartment 3-necked cell with a flat borosilicate window, using an Ivium CompactStat potentiostat. A three-electrode setup was used with a Pt-counter, Ag/AgCl/KCl_{sat} reference, and a $CuCrO_2$-based working electrode (0.25 cm^2 active area). The aqueous Na_2SO_4 electrolyte solution (0.1 M, pH 5.5) was purged with N_2 for 15 minutes prior to measurements. Electrodes were illuminated from the front using a calibrated Newport Oriel solar light simulator (150 W, 100 mW cm^{-2}, AM 1.5G) with an IR water filter and a UQG Optics UV-Filter (λ > 400 nm).

Controlled potential photoelectrolysis (CPPE). CPPE experiments were conducted in triplicate at 0.0 V vs. RHE in an airtight two-compartment electrochemical cell with a flat quartz window and a Nafion membrane divider. The working compartment volume was 12 mL with a gas headspace of 5 mL and the counter compartment contained 4.5 mL solution and a 3.5 mL headspace. Both compartments were purged with 2 % CH_4 in N_2 for 30 minutes prior to electrolysis and the amount of hydrogen determined using gas chromatography (see above). The partial pressure of H_2 was calculated to account for dissolved gas in the solution and this was added to the amount of hydrogen to obtain the Faradaic efficiency.

Incident photon-to-current efficiency measurements. IPCE spectra were recorded in a 3-necked one-compartment cell with a flat borosilicate glass window in N_2-purged aqueous Na_2SO_4 (0.1 M, pH 5.5). A three-electrode setup with a Pt-counter, Ag/AgCl/KCl_{sat} reference, and a $CuCrO_2$-based working electrode (0.25 cm^2 active area) was used. Monochromatic light was supplied using a 300 W Xenon lamp coupled to a monochromator (MSH300, LOT quantum design). The intensity was adjusted to 1.0 mW cm^{-2} for each wavelength and the applied potential maintained at 0.0 V vs. RHE. Triplicate data was acquired for each electrode tested.

Treatment of data. All analytical measurements were performed in triplicate and are given as unweighted mean ± standard deviation (σ) unless otherwise stated. σ of a measured value was calculated using equation (2).

$$\sigma = \sqrt{\frac{\sum(x-\bar{x})^2}{n-1}} \qquad (2)$$

Where n is the number of repeated measurements, x is the value of a single measurement and $\bar{x}$ is the unweighted mean of the measurements. σ was increased to 5 % of $\bar{x}$ in the event that the calculated σ was below this threshold.

Supporting Figures

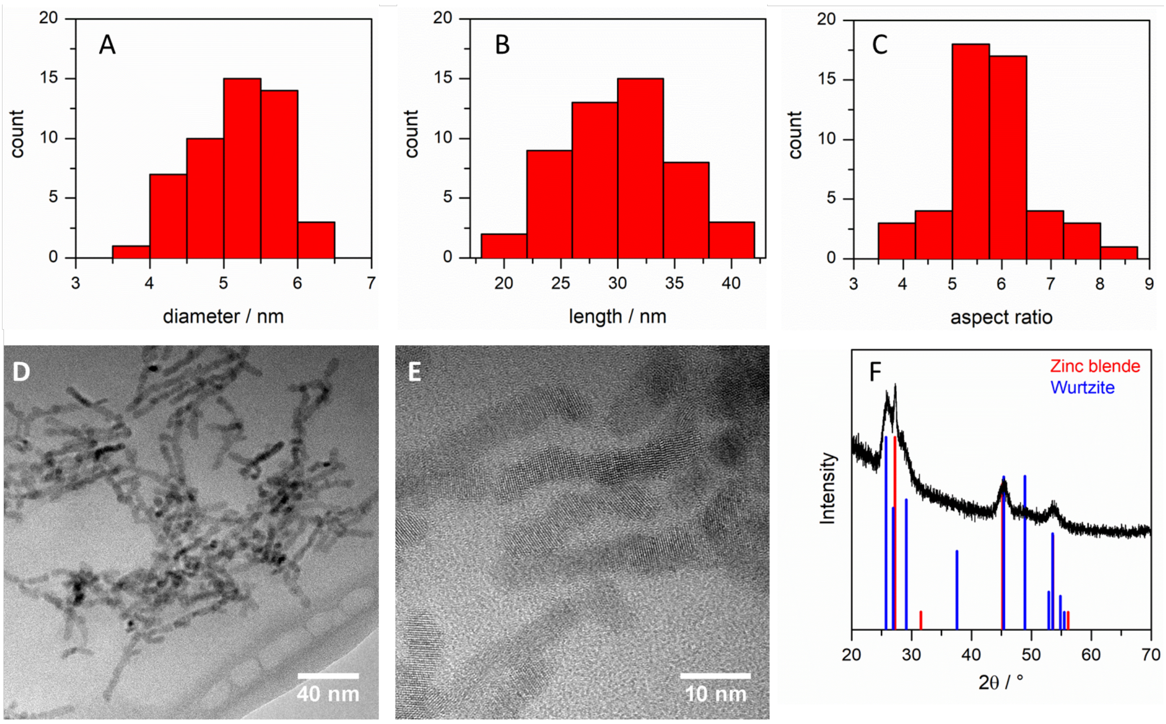

Figure S1. Physical characterization of ligand-free ZnSe nanorods. (A-C) Size distribution from (D-E) transmission electron micrographs; powder X-ray diffractogram with comparison to zinc blende-ZnSe (PDF 01-0715977 37-1463) and Wurtzite-ZnSe (PDF 01-089-2940 15-105).

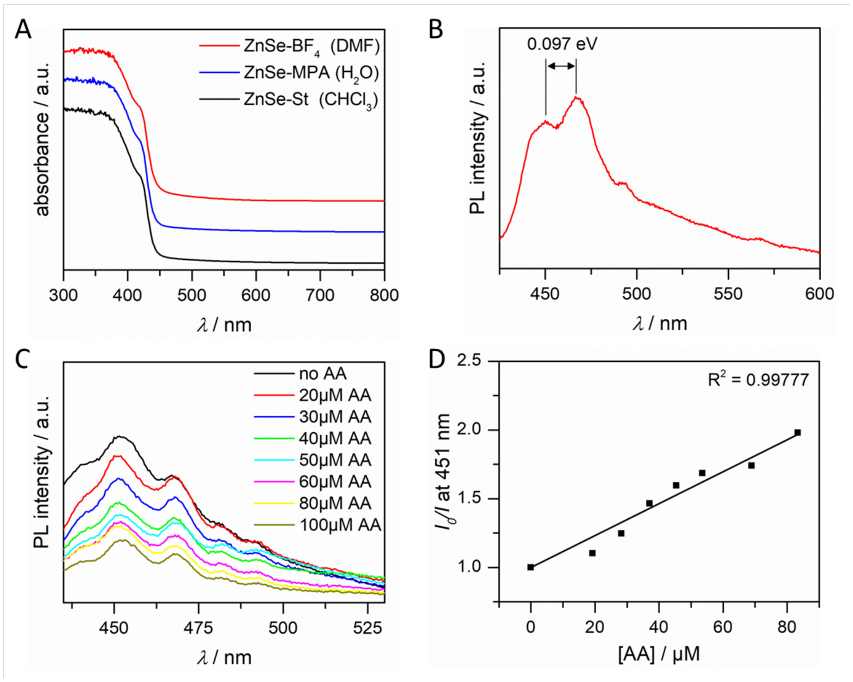

Figure S2. (A) Stacked UV-visible absorption spectra of ZnSe nanorods with different capping ligands. (B) Photoluminescence emission spectrum of ZnSe-BF_4 in DMF with indicated separation of the two emission maxima attributed to Zinc blende (467 nm) and Wurtzite (451 nm) polymorphs (excitation at 370 nm).[4] (C) PL quenching of aqueous ZnSe-BF_4 in the presence of ascorbic acid (AA) at various concentrations (excitation at 390 nm) and (D) corresponding Stern-Volmer plot of the PL intensity at 451 nm.

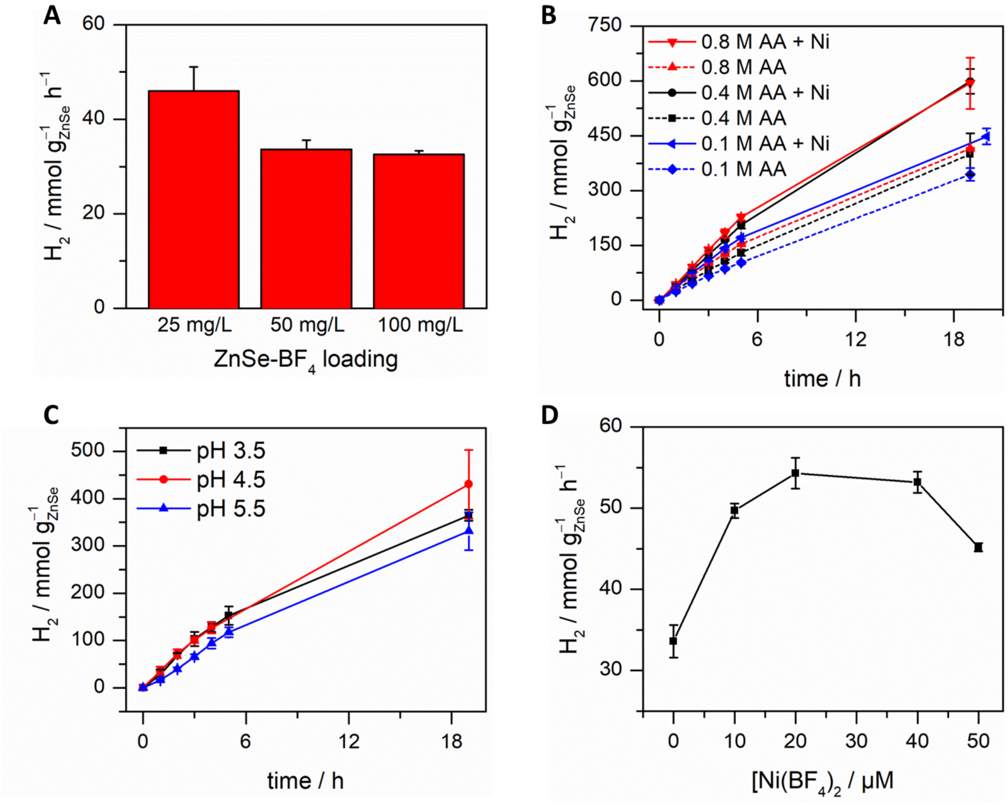

Figure S3. Optimization of the photocatalytic H_2 generation using aqueous ZnSe-BF_4 NRs. (A) Variation of NR concentration (3 h irradiation). (B) Variation of the AA concentration. (C) Variation of the solution pH. (D) Variation of the co-catalyst concentration (3 h irradiation). Conditions unless otherwise stated: 50 mg L^{-1} ZnSe-BF_4, 0.4 M AA, pH 4.5, 25 °C, 100 mW cm^{-2}, AM 1.5G, λ >400 nm, under N_2. Lines between data points have been added to guide the eye.

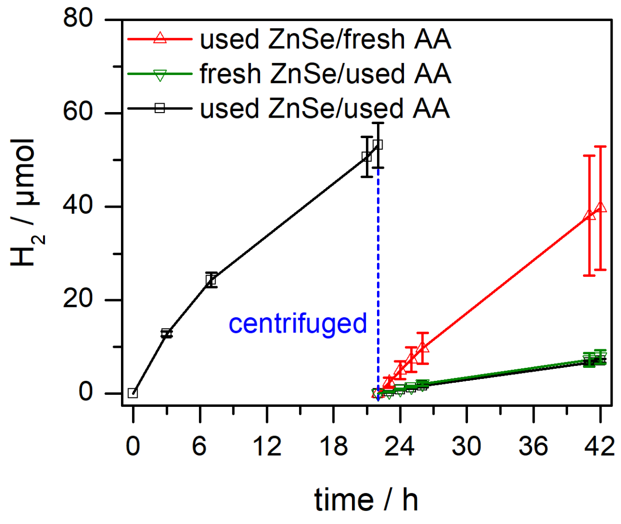

Figure S4. Long-term photocatalytic H_2 generation using aqueous ZnSe-NRs. After 21 h irradiation, samples were centrifuged, and separated precipitate was re-suspended in fresh aq. AA (0.4 M, pH 4.5), or in used aq. AA (supernatant from the same sample), or fresh ZnSe-BF_4 stock solution (6.39 mg ZnSe mL^{-1}, 15.6 µL) was added to used aq. AA (supernatant from another sample). Conditions: 50 mg L^{-1} ZnSe-BF_4, 0.4 M AA, pH 4.5, 25 °C, 100 mW cm^{-2} AM 1.5G, λ >400 nm, under N_2. Lines between data points have been added to guide the eye.

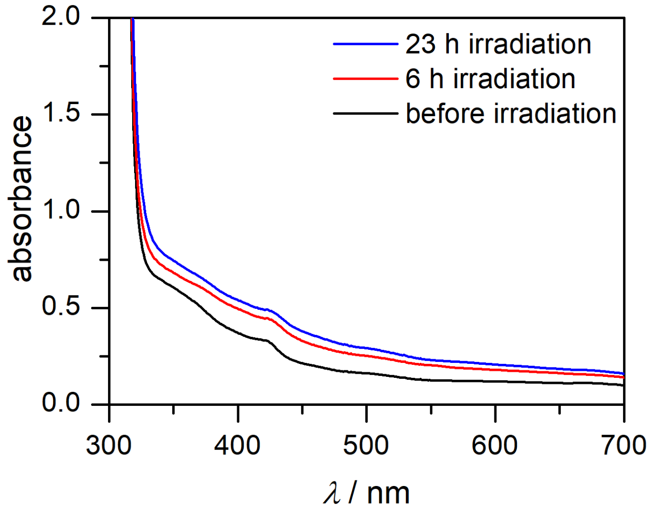

Figure S5. UV-vis spectra of ZnSe-BF_4 NRs during long-term photocatalytic H_2 generation. Conditions: 50 mg L^{-1} ZnSe-BF_4, 20 µM $Ni(BF_4)_2 \cdot 6\,H_2O$, 0.4 M AA, pH 4.5, illuminated with 1.00 mW cm^{-2}, λ = 400±5 nm, under N_2, room temperature.

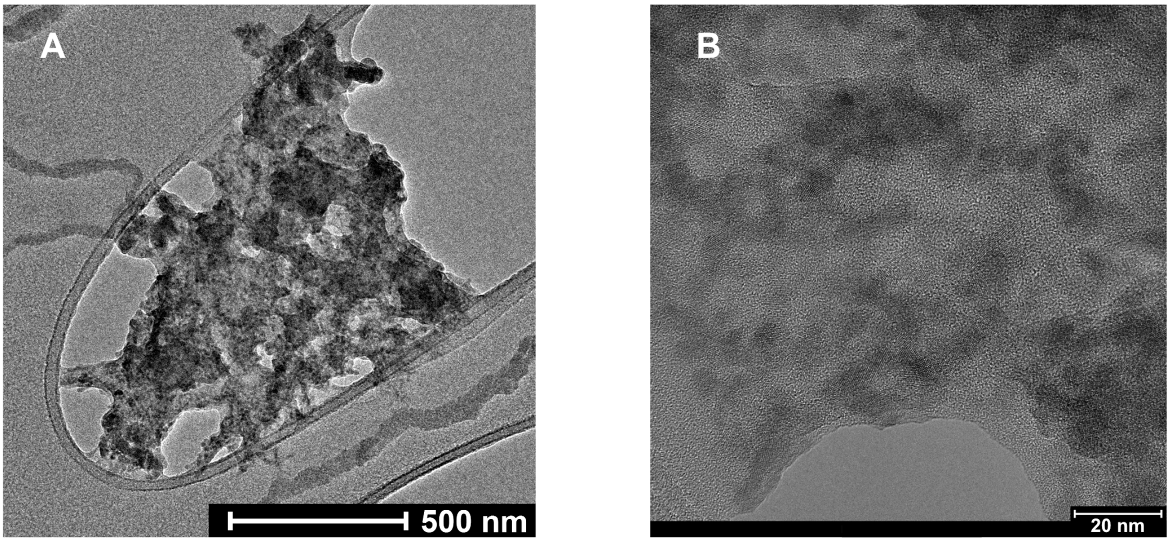

Figure S6. TEM images of ZnSe-BF_4 NRs after 3 hours photocatalytic H_2 generation. Image (A) shows a NR aggregate and (B) confirms the nanocrystalline features which are retained after catalysis. Conditions: 50 mg L^{-1} ZnSe-BF_4, 0.4 M AA, pH 4.5, 25 °C, 100 mW cm^{-2} AM 1.5G, λ >400 nm, under N_2.

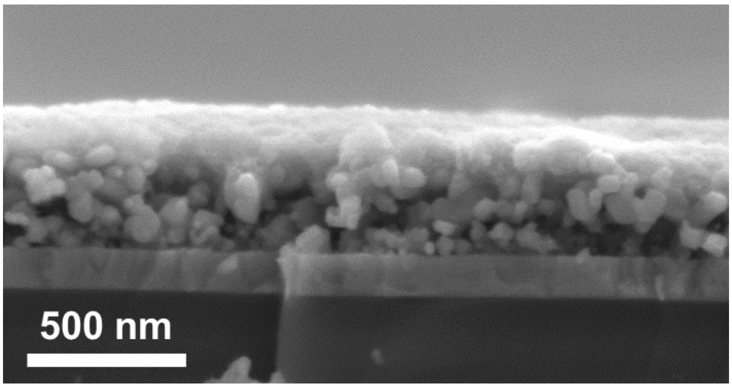

Figure S7. Cross-sectional SEM image of the as-assembled $CuCrO_2$|ZnSe photocathode.

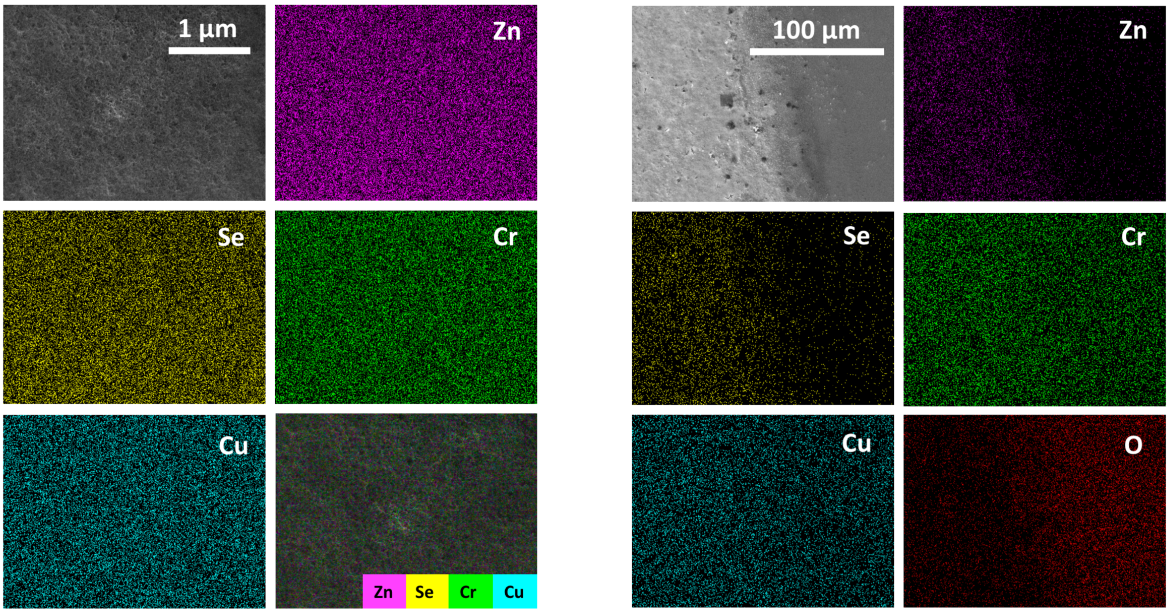

Figure S8. Energy-dispersive X-ray (EDX) spectra of the $CuCrO_2$|ZnSe electrode. Left: Top-down view. Right: View of ZnSe-modified and bare $CuCrO_2$ areas.

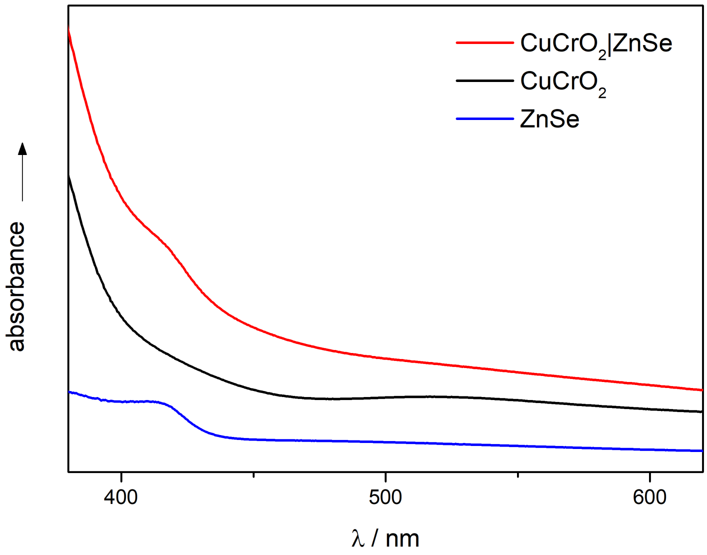

Figure S9. Stacked UV-vis spectra of a $CuCrO_2$ electrode before (black) and after (red) immobilization of ZnSe-BF_4 NRs (blue).

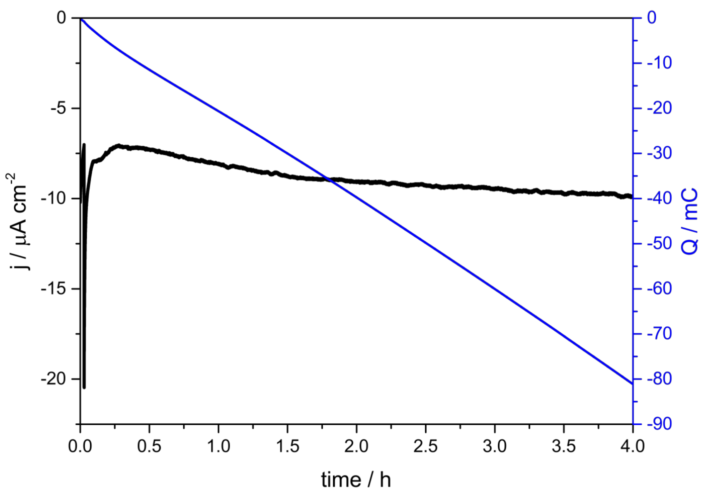

Figure S10. Controlled potential photoelectrolysis (CPPE) of a $CuCrO_2$|ZnSe photocathode (1 cm^2 active geometrical area) held at 0 V vs. RHE. Conditions: aqueous Na_2SO_4 (0.1 M, pH 5.5), illuminated with 100 mW cm^{-2}, AM 1.5G, using a 400 nm cut-off filter, room temperature.

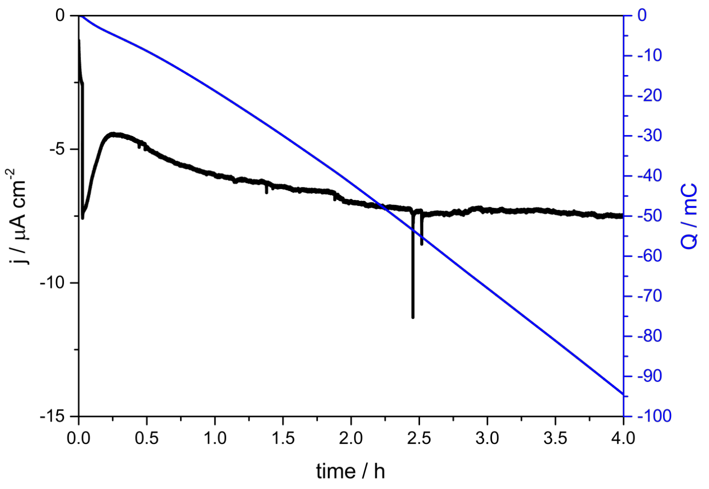

Figure S11. Controlled potential photoelectrolysis of a $CuCrO_2$|ZnSe photocathode (1 cm^2 active area) in the presence of 20 μM $Ni(BF_4)_2$ held at 0 V vs. RHE under visible-light irradiation. Conditions: aqueous Na_2SO_4 (0.1 M, pH 5.5), illuminated with 100 mW cm^{-2}, AM 1.5G, using a 400 nm cut-off filter,room temperature.

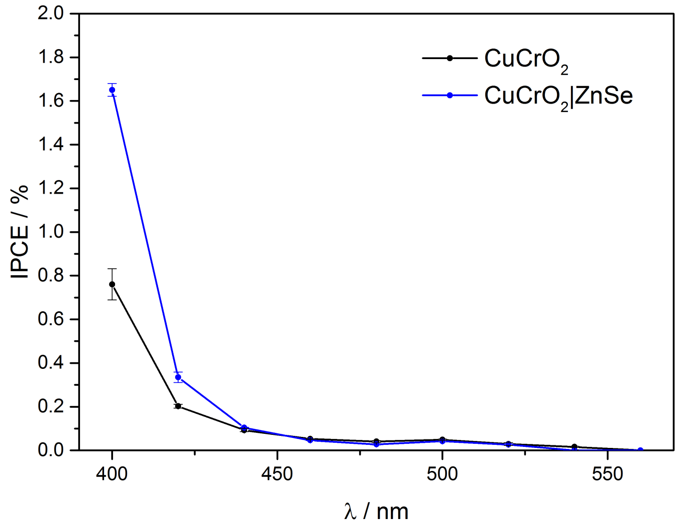

Figure S12. IPCE spectrum for $CuCrO_2$ (black) and $CuCrO_2|ZnSe$ (blue) electrodes recorded in aqueous Na_2SO_4 (0.1 M, pH 5.5) at an applied potential of 0.0 V vs. RHE with light intensity maintained at 1.0 mW cm^{-2}.

Supporting Tables

Table S1. Optimization of the photocatalytic H_2 generation using ZnSe nanorods. Unless otherwise stated, conditions were: 50 mg L^{-1} ZnSe-BF_4, 20 µM $Ni(BF_4)_2 \times 6H_2O$ in 2 mL 0.4 M aq. AA, pH 4.5 under N_2; 3 h irradiation at 100 mW cm^{-2}, AM 1.5G, λ >400 nm, 25 °C).

**different co-catalysts added**			
co-catalyst	c(co-catalyst) / µM	$n(H_2)$ / µmol	activity / $mmol_{H2}\ g_{ZnSe}^{-1}\ h^{-1}$
none	0	10.06±0.60	33.6±2.0
$Fe(BF_4)_2 \times 6H_2O$	20 µM	12.31±1.16	41.0±3.9
$Co(BF_4)_2 \times 6H_2O$	20 µM	13.72±0.38	45.8±1.3
$Ni(BF_4)_2 \times 6H_2O$	20 µM	16.24±0.58	54.3±1.9
K_2PtCl_4	20 µM	0.30±0.16	0.99±0.52
Pt NPs	20 µM	1.50±0.11	4.99±0.36
**varying co-catalyst concentration**			
co-catalyst	c(co-catalyst) / µM	$n(H_2)$ / µmol	activity / $mmol_{H2}\ g_{ZnSe}^{-1}\ h^{-1}$
$Ni(BF_4)_2 \times 6H_2O$	0	10.06±0.60	33.6±2.0
$Ni(BF_4)_2 \times 6H_2O$	10	14.86±0.27	49.7±0.9
$Ni(BF_4)_2 \times 6H_2O$	20	16.24±0.58	54.3±1.9
$Ni(BF_4)_2 \times 6H_2O$	40	15.92±0.39	53.2±1.3
$Ni(BF_4)_2 \times 6H_2O$	50	13.52±0.14	45.2±0.5
**different capping ligands**			
Photocatalyst	co-catalyst	$n(H_2)$ / µmol	activity / $mmol_{H2}\ g_{ZnSe}^{-1}\ h^{-1}$
ZnSe-BF_4	none	10.06±0.60	33.6±2.0
ZnSe-BF_4	20µM $Ni(BF_4)_2 \times 6H_2O$	16.24±0.58	54.3±1.9
ZnSe-MPA	none	6.22±0.16	20.7±0.5
ZnSe-MPA	20 µM $Ni(BF_4)_2 \times 6H_2O$	13.83±0.42	45.9±1.4
ZnSe-St	none	2.19±0.43	7.3±1.4
ZnSe-St	20 µM $Ni(BF_4)_2 \times 6H_2O$	3.64±0.82	12.1±2.7
**varying irradiation spectrum**			
Light spectrum	co-catalyst	$n(H_2)$ / µmol	activity / $mmol_{H2}\ g_{ZnSe}^{-1}\ h^{-1}$
AM 1.5G, >400 nm	none	10.06±0.60	33.6±2.0
AM 1.5G, >400 nm	20 µM $Ni(BF_4)_2 \times 6H_2O$	16.24±0.58	54.3±1.9
AM 1.5G	none	23.06±2.84	76.9±9.5
AM 1.5G	20 µM $Ni(BF_4)_2 \times 6H_2O$	41.16±0.93	137.2±3.1

varying ZnSe concentration			
c(ZnSe-BF_4) / mg_{ZnSe} L^{-1}	co-catalyst	n(H_2) / µmol	activity / $mmol_{H2}$ g_{ZnSe}^{-1} h^{-1}
25	none	6.88±0.76	46.0±5.1
50	none	10.06±0.60	33.6±2.0
100	none	19.47±0.41	32.6±0.7
**varying AA concentration (19 h irradiation)[a]**			
c(AA) / M	co-catalyst	n(H_2) / µmol	activity / $mmol_{H2}$ g_{ZnSe}^{-1} h^{-1}
0.1	none	34.54±1.72	18.1±0.9
0.1	20 µM $Ni(BF_4)_2 \times 6H_2O$	46.82±2.29[b]	22.4±1.1[b]
0.4	none	40.08±5.73	21.0±3.0
0.4	20 µM $Ni(BF_4)_2 \times 6H_2O$	60.03±3.40	31.5±1.8
0.8	none	41.63±0.22	21.9±0.1
0.8	20 µM $Ni(BF_4)_2 \times 6H_2O$	59.52±7.01	31.3±3.7
**varying pH (19 h irradiation)[a]**			
pH	co-catalyst	n(H_2) / µmol	activity / $mmol_{H2}$ g_{ZnSe}^{-1} h^{-1}
3.5	none	36.35±1.14	19.2±0.6
4.5	none	40.08±5.73	22.7±3.8
5.5	none	33.04±3.99	17.4±2.1

[a] Data from two independent experiments; [b] 20 h irradiation.

Table S2. External quantum efficiency (EQE) determination for the photocatalytic H_2 evolution using ZnSe-NRs (50 mg L^{-1} ZnSe-BF_4, in 2 mL 0.4 M aq. AA, pH 4.5 under N_2; I = 1.00±0.01 mW cm^{-2}, A = 0.20 cm^2, λ = 400±5 nm, room temperature).

time / h	$n(H_2)$ / µmol[a]	EQE / %[b]	IQE / %[b]
**no co-catalyst**			
1	0.10±0.03	7.6±2.0	11.8±3.3
2	0.37±0.02	24.1±0.9	36.1±2.4
3	0.68±0.03	26.8±1.1	38.7±1.1
4	0.99±0.04	26.2±1.1	37.7±1.6
5	1.30±0.04	25.8±1.0	36.9±1.6
6	1.60±0.04	24.9±0.5	35.7±0.7
average over hours 3–6		25.9±1.2	37.2±1.7

time / h	$n(H_2)$ / µmol[a]	EQE / %[b]	IQE / %[b]
**with added co-catalyst (20 µM $Ni(BF_4)_2 \times 6\ H_2O$)**			
1	0.10±0.01	8.2±1.2	12.5±1.3
2	0.42±0.03	27.3±1.8	39.2±2.5
3	0.82±0.07	34.0±3.1	48.3±4.1
4	1.25±0.07	36.5±0.5	51.1±0.9
5	1.67±0.08	35.4±1.3	49.3±0.9
6	2.12±0.08	37.5±2.9	52.3±5.0
average over hours 3–6		35.9±2.6	50.2±3.6

[a] Cumulative H_2 measured in headspace.
[b] Quantum efficiency measured per time interval.

Table S3. Control experiments for the photocatalytic H_2 generation using ZnSe-BF_4. Unless otherwise stated, conditions were: 50 mg L^{-1} ZnSe-BF_4, 20 µM $Ni(BF_4)_2 \times 6\ H_2O$ in 2 mL 0.4 M aq. AA, pH 4.5 under N_2; 100 mW cm^{-2}, AM 1.5G, λ >400 nm, 25 °C.

description	time / h	$n(H_2) \pm \sigma$ / µmol
standard experiment	19	72.43±1.75
no $Ni(BF_4)$	19	45.01±2.42
no AA	20	0.015±0.011
no light	21	not detected
no ZnSe-BF_4	19	not detected

Table S4. Determination of ZnSe loading on $CuCrO_2$ using ICP-OES. Conditions: Digestion in 10% v/v HNO_3 in MilliQ® H_2O, 1 cm^{-2} electrode

photoelectrode	Zn^{2+} / µg cm^{-2}	Se^{2-} / µg cm^{-2}
$CuCrO_2$	0.1 ± 0.1	0
$CuCrO_2$ |ZnSe-NRs	6.4 ± 0.3	7.0 ± 0.4

Table S5. Controlled-potential photoelectrolysis using a $CuCrO_2$\|ZnSe photocathode. Conditions: 0.1 M $NaSO_4$ (pH 5.5), E_{app} = 0 V *vs.* RHE, illumination of 100 mW cm^{-2}, AM 1.5G, λ > 400 nm, 1 cm^2 active area.

photoelectrode	time / h	$n(H_2)$[a] / nmol cm^{-2}	F.E. / %
$CuCrO_2$ |ZnSe-NRs (entry 1)	4	38.2	9.0
$CuCrO_2$ |ZnSe-NRs (entry 2)	4	31.9	7.6
$CuCrO_2$ |ZnSe-NRs (entry 3)	4	34.0	5.2
	**average**	**34.7 ± 3.2**	**7.3 ± 1.9**
$CuCrO_2$ |ZnSe-NRs|Ni^{2+} [b]	4	47.8	9.7
$CuCrO_2$ blank	4	not detected	–

[a] corrected for the dissolved H_2 using Henry's law; [b] in presence of 20 µM $Ni(BF_4)_2$

Supporting references

[1] P. Reiss, G. Quemard, S. Carayon, J. Bleuse, F. Chandezon, A. Pron, *Mater. Chem. Phys.* **2004**, *84*, 10-13.

[2] M. F. Kuehnel, C. D. Sahm, G. Neri, J. R. Lee, K. L. Orchard, A. J. Cowan, E. Reisner, *Chem. Sci.* **2018**, *9*, 2501-2509.

[3] C. M. Chang, K. L. Orchard, B. C. M. Martindale, E. Reisner, *J. Mater. Chem. A* **2016**, *4*, 2856-2862.

[4] C.-Y. Yeh, S.-H. Wei, A. Zunger, *Phys. Rev. B* **1994**, *50*, 2715-2718.

End of Supporting Information
